# Analysis of Serum MicroRNAs as Potential Biomarker in Coronary Bifurcation Lesion

**DOI:** 10.1155/2015/351015

**Published:** 2015-04-27

**Authors:** Yan Liu, Shaoliang Chen, Junjie Zhang, Shoujie Shan, Liang Chen, Rong Wang, Jing Kan, Tian Xu

**Affiliations:** Department of Cardiology, Nanjing First Hospital, Nanjing Medical University, Changle Road No. 68, Nanjing, Jiangsu 210029, China

## Abstract

Recent evidence suggests that cell-derived circulating miRNAs may serve as the biomarkers of cardiovascular diseases. However, no study has investigated the potential of circulating miRNAs as biomarker for coronary bifurcation lesion. In this study, we aimed to characterize the miRNA profiles that could distinguish coronary bifurcation lesion and identify potential miRNAs as biomarkers of coronary bifurcation lesion. We employed miRNA microarray to screen serum miRNAs profiles of patients with coronary bifurcation lesion and coronary nonbifurcation lesions. We identified 197 miRNAs differentially expressed, including 150 miRNAs upregulated and 47 miRNAs downregulated. We chose 3 miRNAs with significant differences for further testing in 200 patients. RT-PCR analysis of serum samples confirmed that miR30d was upregulated and miR1246 was downregulated in the serum of coronary bifurcation lesion patients compared with nonbifurcation lesion patients. Our findings suggest that these miRNAs may have a role in the pathogenesis of coronary bifurcation lesion and may represent novel biomarkers for the diagnosis and prognosis of coronary bifurcation lesion.

## 1. Introduction

Coronary artery disease (CAD) is one of the leading causes of death worldwide. The pathogenesis of CAD remains incompletely understood and both genetic and environmental factors are involved in this complex disease [1, 2]. Coronary bifurcation lesion is the most challenging lesion in percutaneous coronary interventional medicine because the rate of restenosis and major adverse cardiac event is significantly higher than nonbifurcation lesion [3]. MicroRNAs (miRNAs) are key regulators of gene expression that have been widely associated with a variety of diseases [4, 5]. Recent evidence suggests that cell-derived circulating miRNAs may serve as the biomarkers of cardiovascular diseases including CAD [6]. However, no study has investigated the potential of circulating miRNAs as biomarker for coronary bifurcation lesion.

In this study, we aimed to characterize the miRNA profiles that could distinguish coronary bifurcation lesion and identify potential miRNAs as biomarkers of coronary bifurcation lesion.

## 2. Methods

### 2.1. Patients

In the matched case-control study, we recruited 5 patients with coronary bifurcation lesion aged 45–87 years (average 72.4 ± 16.4 years) and 5 control patients with coronary nonbifurcation lesion aged 46–75 years (average 59.6 ± 11.7 years). For large sample validation, we recruited 100 patients with coronary bifurcation lesion aged 45–88 years (average 67.8 ± 10.9 years) and 100 control patients with coronary nonbifurcation lesion aged 40–84 years (average 65.5 ± 10.2 years). Serum samples of all subjects were collected.

Coronary bifurcation lesion was defined as a junction of a main vessel and a side branch (with a minimal diameter of 1.5 mm) [7]. Coronary bifurcation lesion patients and control subjects had no other concomitant diseases, including severe cardiomyopathy or valvular heart disease, lung disease, significant cardiac dysfunction or liver and kidney dysfunction, thrombotic disease or blood disease, and connective tissue disease or malignancy or active infection such as hepatitis and tuberculosis.

### 2.2. Serum Samples

The blood was collected from each subject into EDTA anticoagulant tube and centrifuged at 3,000 rpm for 10 min, and the supernatant was stored at −80°C.

### 2.3. RNA Extraction

Total RNA was isolated using TRIzol (Invitrogen) and miRNeasy Mini Kit (QIAGEN) according to the manufacturer's instructions. RNA was dissolved in nuclease-free water by passing a few times through a pipette tip. RNA quality and quantity were measured by using nanodrop spectrophotometer (ND-1000, Nanodrop Technologies) and RNA integrity was determined by gel electrophoresis.

### 2.4. RNA Labeling

RNA was labeled using miRCURY Hy3/Hy5 Power Labeling Kit (Exiqon, Vedbaek, Denmark) according to the manufacturer's guideline. One microgram of each sample was 3′-end-labeled with Hy3TM fluorescent label, using T4 RNA ligase. The mixture was incubated for 30 min at 37°C, and the reaction was terminated by incubation for 5 min at 95°C. Then, 3.0 *μ*L of labeling buffer, 1.5 *μ*L of fluorescent label (Hy3TM), 2.0 *μ*L of DMSO, and 2.0 *μ*L of labeling enzyme were added into the mixture. The labeling mixture was incubated for 1 h at 16°C, and the reaction was terminated by incubation for 15 min at 65°C.

### 2.5. miRNA Microarray

Hy3TM-labeled samples were hybridized on the miRCURYTM LNA Array (v.18.0) (Exiqon) according to the manufacturer's manual. Following the hybridization, the slides were achieved, washed several times using Wash Buffer Kit (Exiqon), and finally dried by centrifugation for 5 min at 400 rpm. Then, the slides were scanned using the Agilent Microarray Scanner (part number G2505C).

### 2.6. Quantitative RT-PCR

Quantitative PCR was performed on an ABI 7500 system (Applied Biosystems, Foster City, CA). RNA was reverse transcribed with the TaqMan miRNA Reverse Transcription Kit (ABI) according to the manufacturer's instructions. Subsequently, 2.5 ul of the product was used for detecting miRNA expression by quantitative polymerase chain reaction with TaqMan miRNA Assay Kits (ABI).

### 2.7. Data Analysis

The intensity of green signal was calculated after background subtraction and four replicated spots of each probe on the same slide have been averaged. We used Median Normalization Method to obtain “Normalized Data,” Normalized  Data = (Foreground − Background)/median; the median was 50 percent quantile of microRNA intensity which was larger than 30 in all samples after background correction. After normalization, the statistical significance of differentially expressed miRNA was analyzed by *t*-test. A threshold cut-off value was set to 1 fold change which indicates that the expression of a given miRNA is uniform in both case and control groups. The fold change of greater than 1 indicated upregulated miRNAs, whereas the fold change of less than 1 indicated downregulated miRNAs. Unsupervised hierarchical clustering and correlation analysis was performed on miRNA data. Data were expressed as mean ± SD and analyzed using SPSS13.0 software. Categorical variables were compared by *χ* ^2^ test, and continuous variables were compared by *t*-test. A value of *P* < 0.05 was considered significant.

## 3. Results

### 3.1. The Characteristics of the Patients

To characterize the miRNAs profiles of coronary bifurcation lesion, we recruited 5 patients with coronary bifurcation lesion and 5 patients with coronary nonbifurcation lesion as the control. The characteristics of these patients were listed in Table 1.

### 3.2. Identification of miRNAs Expression Patterns of Patients with Coronary Bifurcation Lesion

**Figure 1:**
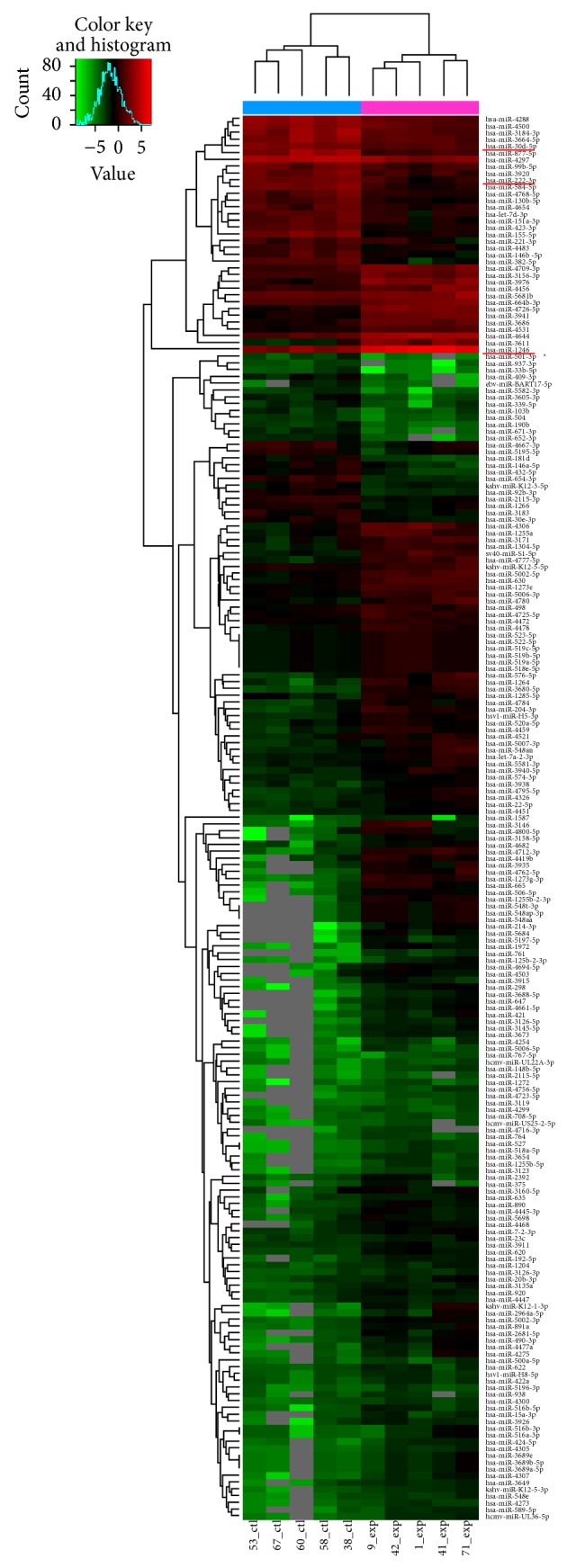
Heat map illustrating the expression patterns of upregulated and downregulated miRNAs in patients with bifurcation lesion. Upregulated miRNAs were indicated by red while downregulated miRNAs were indicated by green. The three candidate miRNA markers miR30d, miR222, and miR1246 were underlined in red.

We performed microarray analysis to identify miRNAs expression patterns of patients with coronary bifurcation lesion. We made a heat map to visualize the results of the two-way hierarchical clustering of miRNAs (Figure 1). The color scale shown at the top illustrated the relative expression level of a miRNA: red represented a high relative expression level while green represented a low relative expression level [8].

**Figure 2:**
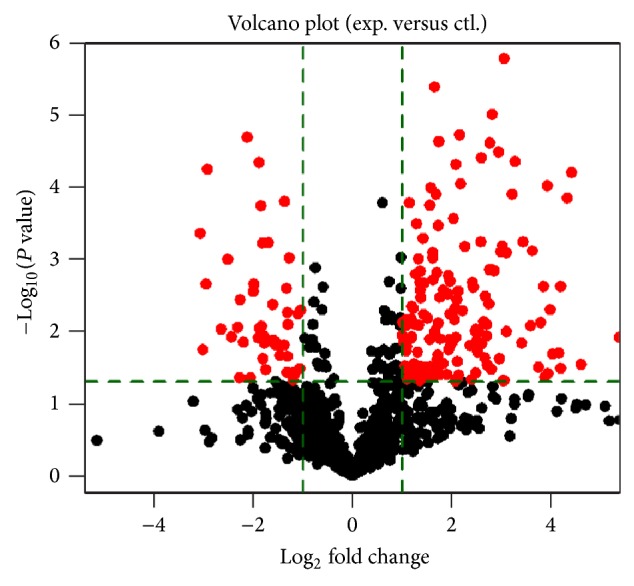
miRNAs differentially expressed in patients with bifurcation lesion and patients with nonbifurcation lesion. The volcano plots illustrated miRNAs differentially expressed: dots in black indicted the miRNAs that did not reach significant changes of expression; dots in red on the left indicated the miRNAs that had significant downregulation of expression; and dots in red on the right indicated the miRNAs that had significant upregulation of expression.

Furthermore, we made a Volcano plot to illustrate miRNAs differentially expressed between coronary bifurcation lesion and coronary nonbifurcation lesion (Figure 2). We identified 197 miRNAs differentially expressed between coronary bifurcation lesion and coronary nonbifurcation lesion, including 150 miRNAs upregulated and 47 miRNAs downregulated (Supplemental File 1 in Supplementary Material available online at http://dx.doi.org/10.1155/2015/351015).

### 3.3. Confirmation of Different miRNAs in Serum of Patients with Coronary Bifurcation Lesion

To confirm our results, we chose three of the most differentially expressed miRNAs, including 2 upregulated (miR30d and miR222) and one downregulated (miR1246), which showed difference by more than 10 times. We enrolled 100 patients with bifurcation lesion and 100 patients with nonbifurcation lesion. As illustrated in Table 2, there were no significant differences in the two groups, including the age, the gender, the status of smoking, hypertension, and diabetes.

**Figure 3:**
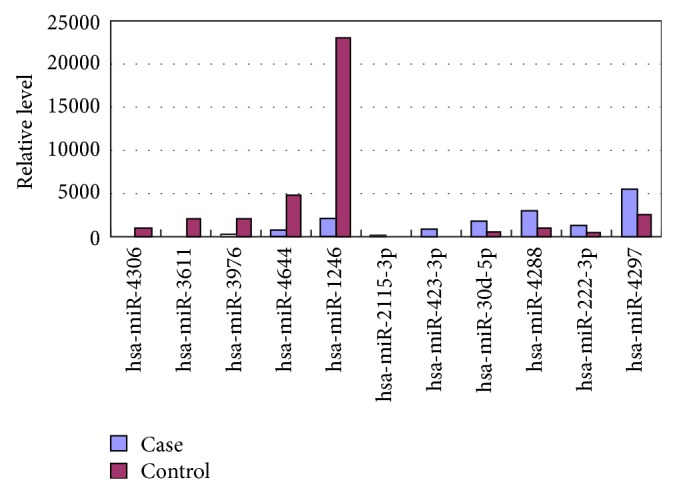
qRT-PCR analysis of several miRNAs differentially expressed in case and control groups. Case: bifurcation lesion patients; control: nonbifurcation lesion patients. Shown were representative data from three independent experiments.

As shown in Figure 3, RT-PCR analysis showed that circulating serum level of miR30d was profoundly elevated in patients with coronary bifurcation lesion compared to nonbifurcation lesion patients (*P* < 0.05). Circulating level of miR222 was modestly but not significantly increased in coronary bifurcation lesion patients compared to nonbifurcation lesion patients (*P* = 0.881). Moreover, serum level of miR1246 was significantly lower in bifurcation lesion patients than in nonbifurcation lesion patients (*P* < 0.05). These data confirmed our results of microarray analysis.

## 4. Discussion

Coronary artery disease (CAD) is a multifactorial disease that can be influenced by a multitude of environmental and heritable risk factors. CAD has serious impact on human life and health. Coronary atherosclerosis is a main reason that causes CAD. Atherosclerosis is a chronic and progressive pathologic process characterized by the accumulation of lipid and fibrous elements in the large arteries, which causes a number of cardiovascular-related diseases. The development of atherosclerosis involves the following steps: foam cell formation, fatty streak accumulation, migration and proliferation of vascular smooth muscle cells (VSMCs), and fibrous cap formation. Finally, the rupture of the unstable fibrous cap causes thrombosis that leads to unstable coronary syndromes, myocardial infarction, and stroke.

Coronary intervention is an effective means of coronary heart disease treatment. Coronary bifurcation is prone to develop atherosclerotic plaque due to turbulent blood flow and high shear stress. Treatment of coronary bifurcation lesion represents a challenging area in interventional cardiology but recent advances in percutaneous coronary interventions (PCI) have led to the dramatic increase in the number of patients successfully treated percutaneously. Compared with nonbifurcation interventions, bifurcation interventions have a lower rate of procedural success, higher procedural costs, longer hospitalization, and a higher clinical and angiographic restenosis. Introduction of drug-eluting stents (DES) has resulted in a lower event rate and reduction of main vessel (MV) restenosis. However, side branch (SB) ostial residual stenosis and long-term restenosis remain a problem [9].

MicroRNAs (miRNAs) are a class of short, noncoding, single stranded RNA molecules, approximately 22 nucleotides in length. They negatively regulate gene expression either through inhibition of mRNA translation or by promoting mRNA degradation. Emerging evidence suggests that miRNAs are pivotal regulators of various processes including cell proliferation, differentiation, apoptosis, survival, motility, and morphogenesis [10]. Recently, specific miRNA expression profiles have been reported as a prognostic factor or a predictive factor for disease progression. In particular, serum miRNAs may be used as a biomarker in diagnosis [6]. In pathological process, miRNAs are linked to myocardial hypertrophy, myocardial fibrosis, heart failure, and arrhythmias. A series of miRNAs are involved in pathological progression of coronary heart disease and play pivotal roles in the development of the disease [11–13].

Hoekstra et al. investigated the potential of miRNAs as biomarkers for CAD and reported that unstable angina pectoris patients could be discriminated from stable patients based on the relatively high expression levels of miR-134, miR-198, and miR-370 in peripheral blood mononuclear cells [14]. Therefore, we hypothesized that in CAD serum miRNA levels could be changed and those miRNAs can be used as the biomarkers. In this study, we performed microarray analysis to identify 197 miRNAs differentially expressed in the serum of patients with coronary bifurcation lesion, including 150 miRNAs upregulated and 47 miRNAs downregulated. After a rigorous selection, we screened 3 miRNAs differentially expressed, including downregulated miR1246 and upregulated miR30d and miR222. Further large sample validation in 200 patients demonstrated a distinct serum miRNA expression pattern in patients with coronary bifurcation lesion; miR30d was upregulated and miR1246 was downregulated compared with nonbifurcation lesion patients. Consistent with our results, a recent study reported that miR30d was upregulated in diabetic cardiomyopathy [15]. Therefore, miR30d may be a promising marker and therapeutic target for various cardiovascular diseases. miR222 has been shown to play an important role in the regulation of vascular inflammation [16]. In this study, we found that circulating level of miR222 was not significantly increased in coronary bifurcation lesion patients compared to nonbifurcation lesion patients. It will be important to further confirm the function of miR222 in coronary bifurcation lesion. Up to now, miR1246 is mainly reported to be involved in cancer development [17, 18]. The role of miR1246 in cardiovascular diseases needs further studies.

## 5. Conclusions

This is the first study using microarray method to investigate the association between serum miRNAs and coronary bifurcation lesion. Evaluation of the screened up- and downregulated miRNAs according to their target mRNAs and biological significance will give some clues for their functional role in coronary bifurcation lesion. Our findings suggest that these miRNAs may have a role in the pathogenesis of coronary bifurcation lesion and may represent novel biomarkers for the diagnosis and prognosis of coronary bifurcation lesion.

## Supplementary Material

The list of miRNAs upregulated and downregulated between coronary bifurcation lesion and coronary nonbifurcation lesion.

## Figures and Tables

**Table 1:** The characteristics of the patients for microarray analysis.

	Coronary bifurcation lesion *n* = 5	Coronary nonbifurcation lesion *n* = 5
Age (years)	72.4 ± 16.4	59.6 ± 11.7
Males	4 (80%)	2 (40%)
Hypertension	4 (80%)	4 (80%)
Diabetes	0 (0%)	0 (0%)
Stroke	2 (40%)	2 (40%)
Current smoker	2 (40%)	2 (40%)
Lesion site LAD	4 (80%)	1 (20%)
Lesion site LM	0 (0%)	4 (80%)
Lesion site RCA	1 (20%)	0 (0%)
Lesion calcification	0 (0%)	3 (60%)
Needing rotablation	0 (0%)	1 (20%)
Restenosis	0 (0%)	1 (20%)
Thrombus	1 (20%)	1 (20%)
TIMI grade 0	3 (60%)	2 (40%)
TIMI grade 1	2 (40%)	3 (60%)
Medina classification 111	0 (0%)	4 (80%)

**Table 2:** Confirmation of differential miRNA expression in 200 patients.

	Bifurcation lesion (*n* = 100)	Nonbifurcation lesion (*n* = 100)	*P*
Age (years)	67.8 ± 10.9	65.5 ± 10.2	0.377
Males	52	56	0.55
Smoker	52	50	0.82
Hypertension	61	60	0.76
Diabetes	28	30	0.51
miR30d level	0.0258 ± 0.0566	0.0017 ± 0.0006	0.000
miR222 level	0.1024 ± 0.0616	0.0953 ± 0.0693	0.881
miR1246 level	0.0346 ± 0.0567	0.3004 ± 0.2469	0.000
